# Electrophoretic Pattern of Serum Proteins and Concentrations of Serum Protein Fractions in Lambs Infected With *Haemonchus contortus*

**DOI:** 10.1155/vmi/5743624

**Published:** 2026-10-28

**Authors:** Csilla Tóthová, Veronika Glembová, Zora Váradyová, Marián Várady, Veronika Oršuľaková, Oskar Nagy

**Affiliations:** ^1^ Clinic of Ruminants University of Veterinary Medicine and Pharmacy in Košice Komenského 73 041 81 Košice Slovak Republic uvm.sk; ^2^ Institute of Animal Physiology of Centre of Biosciences of Slovak Academy of Sciences Šoltésovej 4-6 040 01 Košice Slovak Republic; ^3^ Institute of Parasitology of Slovak Academy of Sciences Hlinkova 3 040 01 Košice Slovak Republic

## Abstract

The aim of this study was to evaluate the blood serum protein profile separated by electrophoresis in 10 lambs experimentally infected with *Haemonchus contortus* and to describe the changes in the distribution of serum protein fractions over a 107‐day postinfection period. These lambs were a part of a previous experiment that examined the effects of alternative treatment on the *H. contortus* infection. Blood samples were collected from 10 lambs infected with *H. contortus* on Days 0 (before infection), 14, 28, 42, 56, 70, 84, 98, and 107 postinfection. Total serum protein concentrations were analyzed using the biuret method, and the major protein fractions were separated with agarose gel electrophoresis. Only one lamb showed clinically observable signs of haemonchosis; these results were excluded from the statistical analysis. Changes in the analyzed parameters in animals without clinical signs (*n* = 9) were highly significant, except for the relative values of β1‐globulins. The most notable changes after infection were observed between Days 14 and 42, where the absolute concentrations of total proteins, albumin, *α*_2_‐, *β*_1_‐, and *γ*_1_‐globulins decreased significantly (*p* < 0.05 to *p* < 0.001). Conversely, the relative concentrations of *α*_1_‐and *α*_2_‐globulins increased (*p* < 0.01 and *p* < 0.001, respectively), and *γ*_1_‐globulin decreased (*p* < 0.01). From Day 56 onward, several parameters showed a steady trend toward baseline values before infection. The changes observed in the lamb with clinical signs were more severe, characterized by extremely low total protein and albumin levels and markedly higher relative values of *α*‐ and *γ*‐globulin fractions on Days 98 and 107 postinfection. These results suggest that alterations in the protein profile and serum protein fraction distribution occur even in animals infected with *H. contortus* without clinical symptoms, which should be considered when addressing this health issue.

## 1. Introduction

Electrophoresis of serum proteins is a key laboratory technique in clinical biochemistry, available for the separation and fractionation of proteins present in the blood serum. In human medicine, one of the most common applications of serum protein electrophoresis is the detection of abnormal proteins [1, 2]. It has also been introduced to veterinary medicine, where it can be primarily used for the identification of clinically significant abnormalities associated with protein metabolism and dysproteinemias (predominantly hyperproteinemia or hyperglobulinemia) or to evaluate the type of inflammation and stimulated humoral immune response [3, 4]. Correctly interpreted results of serum protein electrophoresis may be one of the most useful diagnostic methods for the evaluation of serum protein profile, changes of homeostasis, and follow‐up treatment responses also in ruminants. However, due to major species‐ and breed‐related differences and difficulties in the interpretation of electrophoretograms, serum protein electrophoresis is still an underutilized technique in ruminant medicine.

The electrophoretic pattern of serum proteins in sheep was found to be different from that described in cattle, while differences were observed in the number and size of separated protein fractions [5, 6]. Alterations in the serum protein profile have been observed in small ruminants with several metabolic disorders, as well as infectious and parasitic diseases, including infections with a range of gastrointestinal nematodes [7, 8]. Among these parasites, *Haemonchus contortus* (*H. contortus*) is the most prevalent blood‐feeding nematode in sheep of all ages throughout the world, which adversely affects the health, leading to lower production and even death in severe cases, thus causing tremendous economic losses to the small ruminant industry [9–11]. As these worms ingest blood, high infection levels cause severe blood loss and disorders in the hemopoietic system with concurrent hypoproteinemia [12, 13]. Several studies have been conducted to describe the impact of haemonchosis on hematological and biochemical parameters, especially total serum protein (TP) and albumin concentrations in sheep and goats [14, 15]. Although some further alterations in the serum protein profile were observed in lambs with *H. contortus* infections [16, 17], the changes in the distribution of serum protein fractions over a longer period after the infection are less well described. Furthermore, little is known about the serum protein pattern and its alterations in animals with subclinical haemonchosis showing no obvious clinical signs. The lambs used in the present study were a part of a comprehensive experiment that investigated the effects of alternative treatment of lambs infected with *H. contortus* grazing on a seminatural grassland on the growth parameters, parasitological status, ruminal fermentation, and histopathology [18]. Although haemonchosis commonly occurs as a chronic or subclinical infection in sheep, the majority of earlier studies on metabolic responses were conducted in animals with clinical infection. We hypothesized that also subclinical haemonchosis alters the distribution of blood serum proteins, reducing the concentrations of TPs and albumin and increasing globulins. Our goal was to describe the electrophoretic pattern of blood serum proteins in lambs after experimental infection with *H. contortus*, showing no clinically observable signs of the disease, and to evaluate the time‐dependent changes in the distribution and concentrations of the major serum protein fractions during the prolonged period after infection.

## 2. Materials and Methods

### 2.1. Ethical Approval

The study was conducted in accordance with the guidelines of the national legislation in the Slovak Republic (G.R. 377/2012; Law 39/2007) for the care and use of research animals. The animal studies were approved by the Ethical Committee of the Institute of Parasitology of the Slovak Academy of Sciences [18]. The studies were conducted in accordance with the local legislation and institutional requirements. Written informed consent was obtained from the owners for the participation of their animals in this study. The study was performed in accordance with the ethical standards and guidelines approved by the Institutional Committee on protection of animals used for scientific purposes and complied with the institutional requirements of the Code of Ethics for Scientists (under Directive 74/2019/UVLF). The blood samples from the lambs were collected as per the standard sampling procedure used without any harm to the animals.

### 2.2. Animals and Sample Collection

The study was conducted between May and September on pastures of a small private sheep farm located in Eastern Slovakia. A total of 10 clinically healthy lambs of the Cigája breed (five male and five female) at the age of 3–4 months with an initial body weight of 16.1 ± 2.6 kg were included in the study. The number of animals used in the experiment was assigned according to the VICH GL13 guidelines proposed by the European Medicines Agency. The animals used in this study were the control group from a previously published experiment [18]. Before the start of the experiment, they underwent a 20‐day adaptation period, during which they were dewormed with the recommended dose of albendazole (5 mg/kg body weight; Albendavet 1.9% suspension a.u.v., DIVASA‐FARMAVIC S.A., Barcelona, Spain). Fourteen days after treatment, fecal samples were examined for gastrointestinal parasite egg production, and all animals tested negative, confirming the effectiveness of the deworming procedure prior to the experiment. After the adaptation period, the lambs were placed on a fenced plot consisting of meadow grassland with mixed plant species. Furthermore, the plot had a sheepfold and automated drinking water. Animals were also fed commercial concentrate Mikrop ČOJ (MIKROP, Čebín, Czech Republic) during the entire period of the study [18]. The lambs were infected orally with 5000 third‐stage larvae (L3) of the MHCo1 strain of *H. contortus* susceptible to anthelmintics [19]. The animals were clinically monitored before and during the observation period, and, except one lamb, no changes in clinical condition were observed during the evaluation period. During the entire observation period, the animals were not given antiparasitic drugs. Parasitological monitoring was performed throughout the experimental period. Fecal samples were collected directly from the rectum on D14, D20, D28, D42, D56, D70, D84, D98, and D107 postinfection. The number of *H. contortus* eggs per gram (EPG) of feces was determined using a modified McMaster technique with a sensitivity of 50 EPG [20]. Mean egg counts peaked on D28 postinfection (20,990 ± 4880 EPG) and remained consistently high until the end of the experiment on D107 (5008 ± 10,464 EPG), confirming the establishment and persistence of the experimental infection. Samples of blood for analysis were collected at various stages of the infection along a time course of 107 days. The zero sampling was performed before the experimental infection (D0) and then on Days 14, 28, 42, 56, 70, 84, 98, and 107 postinfection. Blood samples were withdrawn from the jugular vein into Serum Gel Z 4.4 mL serum gel separator tubes with clotting activator (Sarstedt, Nümbrecht, Germany). After the delivery to the laboratory, the blood samples were centrifuged at 4000 g for 15 min, and the separated sera were transferred into Eppendorf tubes. The separated sera were not hemolyzed and lipemic. The aliquots of sera were kept frozen at −20 °C and analyzed within 2 weeks. The serum samples were analyzed for the concentrations of TPs (g/L) and the electrophoretic pattern of serum proteins.

### 2.3. Laboratory Analyses

The biuret method was applied to measure the TP concentrations using commercially available diagnostic kits (Randox, Crumlin, United Kingdom) and the automated chemistry analyzer RxMonaco (Randox, Crumlin, United Kingdom). The separation and distribution of serum protein fractions was performed by zone electrophoresis on agarose gel using an automated electrophoresis system, Hydrasys, with commercial diagnostic kits, Hydragel 7 Proteine (Sebia Corporate, Lisses, Evry Cedex, France), according to the application instructions of the manufacturer. Serum protein fractions were separated by zone electrophoresis on a buffered agarose gel at pH 8.8. Ten microliters of each serum sample were applied to perform numbered sample wells on the agarose gel. Control serum (Control Serum Human Normal, Sebia Corporate, France) was included in each run of samples. The electrophoretic migration was performed for 15 min at 20°C constantly at 10 W, 40 mA, and 240 V. After migration, the gels were stained in amidoblack staining solution and then destained with acidic solutions and dried completely [21]. After separation and staining of the gels, the electrophoretic curves were displayed. The staining intensity of individual protein bands was quantified using a densitometer, Epson Perfection V700 (Epson America Inc., California, USA), and the image analysis software Phoresis Version 5.50 (Sebia Corporate, France). The protein fractions were expressed as relative values (%) according to the optical density, and their absolute concentrations (g/L) were quantified from the TP concentrations. Albumin–globulin ratios (A/G) were calculated as well.

### 2.4. Statistical Analysis

Data were submitted to descriptive statistics using the computer program GraphPad Prism V5.02 (GraphPad Software Inc., California, USA). All results were presented as mean and standard deviation (SD). The Kolmogorov–Smirnov test for normality was applied to evaluate the distribution of the data. The overall variances among the results were estimated using the repeated measures ANOVA test, and the Tukey multiple comparison test was applied to test the significance of differences between the sample collections. Differences were considered significant when the *p* value was less than 0.05.

## 3. Results

Clinical examination showed no changes in the clinical condition in 9 out of 10 lambs during the observation period. One animal clinical finding was from the middle of the monitored period, characterized by the presence of mild weakness, pale mucous membranes, and submandibular edema. The results of serum protein analysis from this animal (Table 1, Figure 1a–c) were evaluated and compared separately with the results recorded in the group of animals without clinical signs (Tables 2 and 3, Figures 2a–c and 3a–h).

**TABLE 1:** Changes in relative (%) and absolute values (g/L) of total protein concentrations, monitored serum protein fractions, and albumin‐to‐globulin ratio (A/G) in lamb with clinical signs of anemia during the monitored period.

Variables		Time of sampling (days)								
		0	14	28	42	56	70	84	98	107
Total proteins	g/L	74.8	60.1	51.6	49.6	52.5	45.7	40.4	30.4	28.8

Albumin	%	57.0	51.7	52.0	48.3	53.1	45.2	41.2	26.2	22.9
	g/L	42.6	31.0	26.8	23.9	27.9	20.6	16.6	8.0	6.6

*α*_1_‐globulins	%	6.7	6.7	8.9	9.7	8.6	12.0	12.8	11.4	12.6
	g/L	5.0	4.0	4.6	4.8	4.5	5.5	5.2	3.5	3.6

*α*_2_‐globulins	%	11.8	11.8	14.1	14.3	14.0	15.9	15.9	21.2	21.9
	g/L	8.8	7.1	7.3	7.1	7.3	7.3	6.4	6.4	6.3

*β*_1_‐globulins	%	2.4	2.6	3.5	3.3	3.5	4.0	5.9	7.2	6.1
	g/L	1.8	1.6	1.8	1.6	1.8	1.8	2.4	2.2	1.8

*β*_2_‐globulins	%	2.5	3.9	2.7	3.9	2.3	3.5	2.4	2.9	2.0
	g/L	1.9	2.3	1.4	1.9	1.2	1.6	1.0	0.9	0.6

*γ*_1_‐globulins	%	16.8	19.3	16.3	17.4	16.1	16.2	18.9	26.5	29.3
	g/L	12.6	11.6	8.4	8.6	8.4	7.4	7.6	8.1	8.4

*γ*_2_‐globulins	%	2.8	4.0	2.5	3.1	2.4	3.2	2.9	4.6	5.2
	g/L	2.1	2.4	1.3	1.5	1.3	1.5	1.2	1.4	1.5

A/G ratio		1.33	1.07	1.08	0.93	1.13	0.82	0.7	0.36	0.3

**FIGURE 1:**
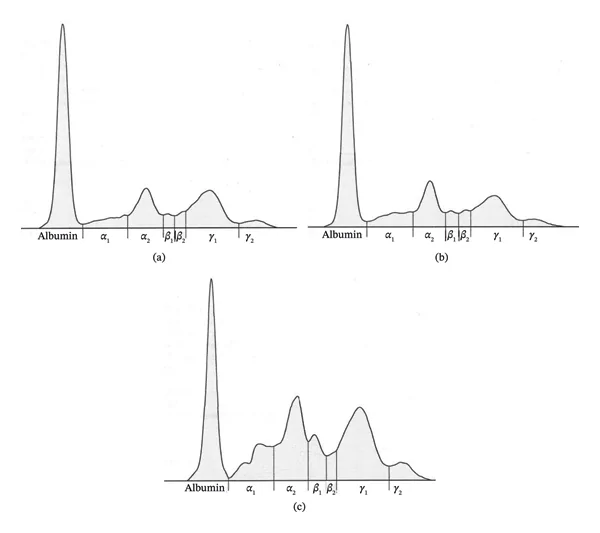
Electrophoretograms characterizing the changes in serum protein fractions before infection–Day 0 (a) during–Day 28 (b) and at the end of the monitored period–Day 107 (c) in animal with the described clinical signs of haemonchosis.

**TABLE 2:** Changes in relative values of concentration of the evaluated serum protein fractions (%) and albumin‐to‐globulin ratio (A/G) in sheep without clinical signs of anemia/*n* = 9) during the monitored period.

Variables		Time of sampling (days)									*p*
		0	14	28	42	56	70	84	98	107	
Albumin	x	54.8^a^	58.2	54.9^ab^	55.5	57.3	57.1	58.2	58.9^b^	59.4^c^	0.0012
	SD	2.9	3.3	5.7	4.0	3.8	4.8	6.2	7.1	7.7	

*α*_1_‐globulins	x	6.23^a^	6.54^ab^	8.19^bc^	7.70	7.31	7.06	6.64^ac^	6.50^ac^	6.46^ac^	< 0.0001
	SD	0.50	0.72	1.32	1.24	1.44	1.41	1.77	1.80	1.83	

*α*_2_‐globulins	x	14.4	13.8^a^	15.3^b^	15.0^abc^	14.5^abcd^	14.0^cd^	13.8^cd^	13.3^e^	13.5^de^	< 0.0001
	SD	0.72	1.0	1.43	1.26	1.03	1.42	1.66	2.16	2.15	

*β*_1_‐globulins	x	3.76	3.48	3.61	3.94	4.03	4.16	4.27	4.06	4.37	0.1202
	SD	0.71	0.69	0.74	0.67	1.29	0.66	0.82	1.01	1.23	

*β*_2_‐globulins	x	3.26^a^	2.83	2.92	2.77	2.70	2.84	2.59	2.66	2.32^b^	0.0382
	SD	0.78	0.44	0.77	0.80	0.84	0.74	0.71	0.91	0.69	

*γ*_1_‐globulins	x	15.1^a^	12.9^b^	13.0^b^	13.2^b^	12.8^b^	12.9^b^	13.1^b^	13.1^b^	12.3^b^	0.0003
	SD	2.19	2.13	2.54	2.19	2.18	2.20	2.66	2.83	2.64	

*γ*_2_‐globulins	x	2.43^a^	2.26^ac^	2.14	2.14^ac^	1.71	1.90	1.40^b^	1.52^bc^	1.67	< 0.0001
	SD	0.95	1.10	1.08	0.38	0.27	0.50	0.27	0.47	0.33	

A/G ratio	x	1.22^a^	1.41	1.25^a^	1.26^a^	1.36	1.37	1.44	1.49^b^	1.54^b^	0.0002
	SD	0.15	0.19	0.27	0.19	0.21	0.25	0.35	0.41	0.45	

*Note:* x, mean values; *p*—significance of changes during the monitored period (ANOVA). a, b, c, d, e—superscripts indicate the significance of the differences in values between the observed blood sampling periods (Tukey test).

Abbreviation: SD, standard deviation.

**TABLE 3:** Changes in total protein concentration and absolute concentrations of the evaluated serum protein fractions (g/L) in sheep without clinical signs of anemia (*n* = 9) during the monitored period.

Variables		Time of sampling (days)									*p*
		0	14	28	42	56	70	84	98	107	
Total proteins	x	70.7^a^	52.0^bc^	51.9^bc^	50.3^b^	53.6^bc^	57.5	61.3^c^	59.6	61.8^c^	< 0.0001
	SD	4.0	3.2	4.4	5.7	6.6	6.6	7.7	8.9	8.7	

Albumin	x	38.6^ac^	30.3^bcd^	28.6^bd^	27.9^d^	30.7	32.8	35.8^bc^	35.3^bc^	37.0^c^	< 0.0001
	SD	2.3	2.2	4.4	4.1	4.5	5.8	6.6	7.3	7.8	

*α*_1_‐globulins	x	4.39^a^	3.39^b^	4.23^ac^	3.87	3.86	3.99	3.99	3.74^bc^	3.87	0.0002
	SD	0.29	0.38	0.53	0.42	0.59	0.53	0.69	0.62	0.66	

*α*_2_‐globulins	x	10.11^a^	7.14^b^	7.89^cd^	7.52^bc^	7.73^bcd^	8.00^cd^	8.36^d^	7.78^bcd^	8.21^d^	< 0.0001
	SD	0.65	0.59	0.71	0.81	0.69	0.69	0.79	0.87	0.92	

*β*_1_‐globulins	x	2.66^a^	1.81^c^	1.84^bc^	1.98^bcd^	1.97	2.39	2.59^d^	2.40	2.66	< 0.0001
	SD	0.52	0.38	0.28	0.24	0.42	0.41	0.43	0.75	0.74	

*β*_2_‐globulins	x	2.30^a^	1.46^b^	1.51	1.40^b^	1.47^b^	1.61	1.59	1.56	1.41^b^	0.0061
	SD	0.57	0.21	0.38	0.44	0.52	0.39	0.47	0.44	0.39	

*γ*_1_‐globulins	x	10.74^a^	6.73^b^	6.78^b^	6.67^b^	6.92^b^	7.43	8.03	7.84	7.58	< 0.0001
	SD	2.07	1.42	1.58	1.55	1.81	1.59	2.14	2.34	2.13	

*γ*_2_‐globulins	x	1.71^a^	1.17	1.10^b^	1.02	0.93^b^	1.09	0.88^b^	0.89^b^	1.08	0.0002
	SD	0.62	0.55	0.48	0.21	0.21	0.27	0.22	0.31	0.24	

*Note:* x, mean values. *p*—significance of changes during the monitored period (ANOVA). a, b, c, d—superscripts indicate the significance of the differences in values between the observed blood sampling periods (Tukey test).

Abbreviation: SD, standard deviation.

**FIGURE 2:**
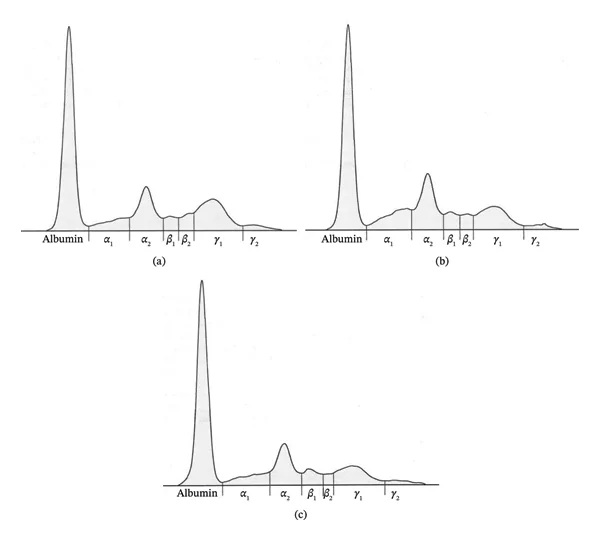
Electrophoretograms characterizing the changes in serum protein fractions before infection–Day 0 (a) during–Day 28 (b) and at the end of the monitored period–Day 107 (c) in animal without clinical signs of haemonchosis.

**FIGURE 3:**
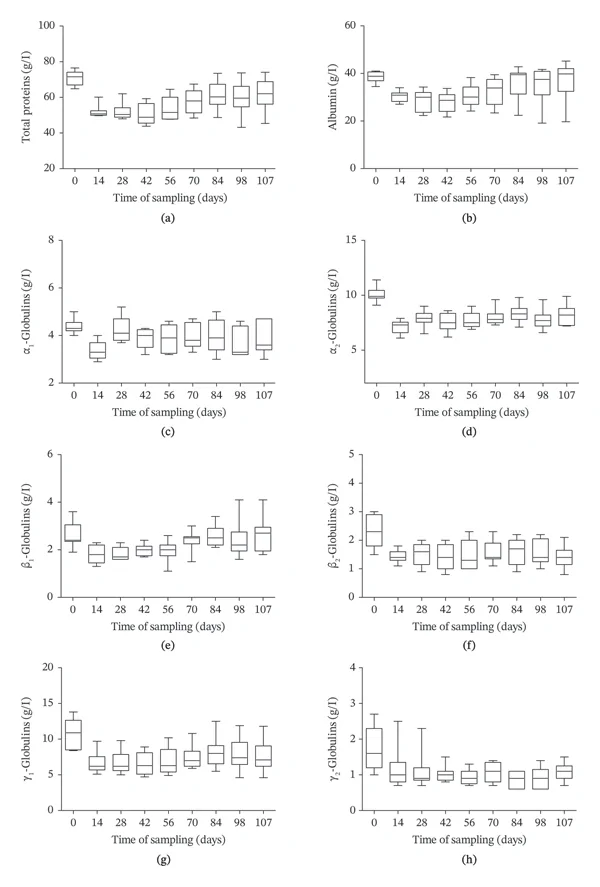
The distribution of the concentrations of total proteins (a), albumin (b), *α*_1_‐ (c), *α*_2_‐ (d), *β*_1_‐ (e), *β*_2_‐ (f), *γ*_1_‐ (g), and *γ*_2_‐globulins (h) in lambs with haemonchosis during the evaluated period. The plots show the median (line within the box), the 25th and 75th percentiles (box), and minimal and maximal values (whiskers).

Seven protein fractions were identified on the serum protein electrophoretogram of the lambs, including albumin, *α*_1_‐, *α*_2_‐, *β*_1_‐, *β*_2_‐, *γ*_1_‐and *γ*_2_‐globulins. The size and shape of separated serum protein fractions differed according to the stage of the pre‐ and postinfection period (Figures 1a–c and 2a–c). Albumin was observable as a high and narrow peak on the anodal side of the electrophoretogram. The *α*‐globulins migrated in *α*_1_‐and *α*_2_‐subfractions. The *α*_1_‐globulin fraction was presented as a lower peak, while the *α*_2_‐globulin fraction was characterized by moderately higher electrophoretic amplitude. The *β*‐globulin fraction was also divided into two smaller subfractions: *β*_1_‐and *β*_2_‐globulins. Two subfractions were also detected in the *γ*‐globulin zone (*γ*_1_ and *γ*_2_), from which the *γ*_1_‐globulin fraction was characterized as a broader and higher peak. In some lambs, there was no clear demarcation between the *β*_2_‐and *γ*_1_‐globulin fractions. In the lamb with clinically observable signs of haemonchosis, a significantly different electrophoretogram was recorded. A clear difference can be observed in most globulin fractions, the ratio of which, except of the *β*_2_‐globulin fraction, is significantly higher at the end of the observed period (Figures 1c and 2c).

Significant changes throughout the evaluated period were shown in the relative concentrations of albumin (Table 2) in lambs without clinical signs of anemia (*p* = 0.0012). After an initial increase, a decrease in the relative values of albumin was noted on the 28th day of observation, followed by a gradual significant increase until the end of the observation period. Of the separated globulin fractions, significant changes in the relative values were found except for the *β*_1_‐fraction in all other monitored fractions (from *p* = 0.0382 to *p* < 0.0001). In the relative concentrations of *α*_1_‐globulins, a significant increase in values was observed on Day 28 postinfection (*p* < 0.05), compared to the pre‐infection period. From Day 42 postinfection, a slight gradual decrease of relative values was observed till the end of the evaluated period. Similarly, an increase in the relative concentrations of *α*_2_‐globulins was shown on Days 28 and 42, with a subsequent gradual decrease in values toward the end of the assessed period. Changes in the relative values of *β*_1_‐globulins were not significant, but the values of this fraction were slightly higher in the second half of the evaluated period. An opposite trend was found in the relative concentrations of *β*_2_‐globulins, with the significantly highest mean value before the infection and the lowest on Day 107 postinfection (*p* < 0.01). Significant changes during the evaluated period were found in the relative concentrations of both γ‐globulin fractions (*p* = 0.0003 and *p* < 0.0001, respectively). The highest average values of both fractions were found before infection. An opposite trend of changes was observed in the A/G, with higher values observed in the postinfection period (*p* = 0.0002).

The evaluation of the absolute concentrations of the analyzed parameters is presented in Table 3 and Figure 3a–h. Highly significant changes during the evaluated period were shown in the concentrations of TPs in lambs after infection with *H. contortus* (*p* < 0.0001). The significantly highest mean TP concentration was recorded before the infection. A significant decrease in values was observed already 14 days postinfection (*p* < 0.0001), and low mean TP concentrations were observed up to 56 days after infection. Subsequently, a gradual increase in TP concentrations was observed until the end of the monitored period. The total protein concentrations were significantly lower toward the end of the observation period compared to the period before infection. Similar highly significant changes were observed in the absolute concentrations of albumin (*p* < 0.0001). The highest concentrations were observed before the infection; a significant decrease in the concentrations of albumin was found shortly after the infection (Days 14–56). Then an increase in albumin concentration was detected and the average value at the end of the observation period was only slightly lower than before infection. Significant variations during the evaluated period were also observed in the absolute concentrations of *α*_1_‐and *α*_2_‐globulins (*p* = 0.0002 and *p* < 0.001, respectively), with a significant decrease in values 14 days after the infection compared to the mean value recorded before the infection. Subsequently, the concentrations of both fractions increased, but they were significantly lower, especially in the *α*_2_‐globulin fraction, than before infection. The absolute concentrations of *β*_1_‐globulins decreased significantly shortly after the infection and were significantly lower until the follow‐up period of 56 days after the infection and then the values increased to the mean value recorded before the infection (Table 3). A significant decrease in values 14 days after the infection was recorded also in the absolute concentrations of *β*_2_‐globulins. Compared to the *β*_1_‐globulin fraction, the concentration of this fraction did not show a significant increase in values in the following period. These changes of concentrations found in both fractions were significant (*p* < 0.0001 and *p* = 0.0061, respectively). A similar significant trend of changes was found for both γ‐globulin fractions (*p* < 0.0001 and *p* = 0.0002, respectively). The values of *γ*_1_‐globulins decreased significantly shortly after the infection (Day 14) (*p* < 0.05) and lower concentrations were recorded in samples collected between 14 and 56 days after infection. At the end of the observed period, the values of the fraction were lower than before infection. The decrease in the absolute concentrations of *γ*_2_‐globulins was detected already at 14 days after infection and the concentrations of this fraction remained lower until the end of the observed period.

Compared to the mean values observed in animals without clinical signs, in the lamb with clinically observable signs of haemonchosis (Table 1), similar trends of changes after infection were noted up to the sampling period of 56 days after infection. After this period, however, markedly different trends of changes in absolute and relative values were found for most of the monitored parameters. The concentrations of TPs and albumin decreased after the infection, but the values did not start to increase again and continued to decrease to extremely low TP (28.8 g/L) and albumin concentrations (6.6 g/L) at the end of the evaluated period. Compared to animals without clinical signs, lower concentrations were also found in *α*‐ and *β*‐globulin fractions, but higher in *γ*‐globulin fractions. Changes in the ratio of individual protein fractions were reflected in different values of absolute concentrations of the above parameters. A marked continuing decrease in the proportion of albumin in the serum relative to globulins (mainly *α*_1_, *β*_1_ and *γ*_1_) was observed after 56 days of infection. This also resulted in a different trend in the A/G ratio.

## 4. Discussion

Natural infections with various gastrointestinal nematodes are common diseases of small ruminants that can cause important metabolic changes and deviations from normal physiology, including the serum biochemistry of infected animals, comprising the protein profile [22]. Several studies have been conducted to date on the impact of gastrointestinal parasitosis on selected biochemical indicators of protein metabolism, most of which have focused mainly on the determination of TP and albumin concentrations with indirect determination of the globulin fraction [13, 23–25]. Abnormalities in the biochemical profile depend not only on the type of parasite and the extent of damage but also on the severity of the infection itself [22, 26]. These changes may not always be accompanied by changes in TP concentrations, as their presence and nature depend on the stage of the disease and the intensity of the damage. The infection with the gastrointestinal worm *H. contortus* in sheep is primarily manifested by clinical signs associated with anemia, as a result of the blood‐sucking activity of the helminth, which in severe cases may lead to death. However, the intensity of changes in the affected animals depends on the degree of infection, the response of the immune system, and the activation of hematopoiesis of the host [27]. Thus, the infection must not always manifest with clinically observable signs and can often remain clinically unrecognized. The results of our study showed changes in the serum protein concentrations and distribution of serum protein fractions, also in animals without clinical signs of haemonchosis. The significant decrease of total protein concentrations from Day 14 after the infection and the lowest values between Days 28 and 42 postinfection may be attributed to loss of blood caused by the blood‐sucking behavior of the parasite from capillaries in the abomasum, loss of large quantities of proteins into the abomasal lumen through disrupted intercellular junctions, tissue damage, and increased gut permeability with subsequent protein‐losing enteropathy [28–31]. Parmar et al. [32] recorded also a decrease of total serum concentrations in sheep infected with 10,000 L3 *H. contortus* already from the second week after the infection, while the decrease of values was statistically significant from Day 28 postinfection. Similarly, Bordoloi et al. [13] found in infected sheep reduced TP concentration from Day 28 postinfection with a further slight decrease till Day 42 postinfection. Decreased TP concentrations in sheep with haemonchosis were also observed by Ahmad et al. [33] and Jaheed et al. [25], as well as Malede et al. [34]. In our long‐lasting study, after the critical period between Days 14 and 42 postinfection, a repeated, slight, gradual increase of TP values was observed, which may reflect possible adaptation of the organism to the presence of parasites with a mild restoration and reparation of impaired functions. On the other hand, the TP concentration in the lamb with clinical signs of haemonchosis showed a further decrease of values, with the lowest concentration on Day 107 postinfection (28.8 g/L).

Very similar, highly significant decrease already from Day 14 after the infection was observed in the concentrations of albumin due to selective loss of albumin from the blood vessels and through the damaged intestinal mucosa, which is smaller in size and osmotic sensitivity to fluid movement [35, 36]. Similarly, Parmar et al. [32] described in sheep a decrease of albumin concentrations from the second week after the infection with *H. contortus*, and Bordoloi et al. [13] observed a significant decrease of albumin values already from Day 7 till Day 42 after the infection. A marked reduction in albumin concentrations in sheep infected with *H. contortus* was also found by Ahmad et al. [16, 33] and Alam et al. [15], as well as Malede et al. [34]. Similarly to TPs, the albumin concentrations began to increase until Day 107 postinfection, when they reached concentrations comparable to pre‐infection values. According to Friedriks et al. [37], when serum albumin concentrations decrease markedly (typically below 15 g/L), the intravascular colloid osmotic pressure decreases significantly, and the fluid from the blood vessels leaks out and accumulates in the surrounding interstitial tissues. This was observable in our study in the lamb with clinical signs of haemonchosis with extremely low albumin concentration observed 107 days after the infection (6.6 g/L).

Regarding the globulin concentrations, most studies evaluated the total globulin concentrations, which is only a calculated value. Our study showed alterations also in the distribution of several globulin fractions, with the most intense changes in the period between Days 14 and 42 postinfection. The relative concentrations of *α*_1_‐and *α*_2_‐globulins in lambs increased significantly after the infection, with the highest percentage on Day 28 postinfection. Fausto et al. [17] observed in untreated lambs infected with *H. contortus* no marked changes in the concentrations of *α*‐globulins throughout the study period of 80 days postinfection. On the other hand, in goats infected with *H. contortus*, a marked increase of *α*‐globulins was recorded by Diogenes et al. [22], but the immune responses against parasitic nematodes in sheep may differ from those in goats and are not well described [38]. Seeing that gastrointestinal parasites, including *H. contortus,* usually cause not only local inflammation of the intestine, tissue injury, and damage to the mucosal structures of the abomasum but may also induce general metabolic disturbances and inflammatory responses, the increased proportion of *α*‐globulins observed in our study in lambs after the infection may be related to the increased synthesis of acute phase proteins belonging to these fractions. Gänheim et al. [39] described no marked increase in the concentrations of haptoglobin in sheep with common gastrointestinal nematode infections. On the other hand, Zhong et al. [40] observed approximately a 10‐ to 30‐fold increase of haptoglobin concentrations in sheep experimentally infected with *H. contortus*, with the strongest increase of values on the 3rd and 28th days postinfection. In the study conducted by Iliev et al. [41], the concentrations of haptoglobin increased considerably as early as the beginning of the experimental infection on Day 4 and peaked on the 7th and the 14th day postinfection. The values were almost doubled and correspond to the growth of larvae and more severe mechanical damage of the abomasal mucosa. The effects of gastrointestinal nematodes on the concentrations of serum amyloid A have not been extensively studied. Although Ulutaş et al. [42] reported an increase in serum amyloid A concentrations in sheep with mixed gastrointestinal nematode infections, the response of serum amyloid A to the experimental infection with *H. contortus* evaluated by Váradyová et al. [43] was weak and non‐uniform. The relative concentrations of *α*‐globulins in the infected lambs from our study started to gradually decrease after peaking on Day 42 postinfection and returned to values before the infection. These changes suggest restoration of homeostasis in the infected animals and adaptation to the parasitic infection. On the other hand, persistently high *α*_1_‐and *α*_2_‐globulin relative concentrations with further marked increase from Day 70 postinfection were detected in the lamb with clinical signs of haemonchosis, which appeared on Day 98 postinfection.

The alterations observed in the *β*‐globulin fractions in the infected lambs were less marked compared to *α*‐globulins. While the relative concentrations of *β*_1_‐globulins increased slightly throughout the postinfection period, the values of *β*_2_‐globulins decreased after the infection. Some further important inflammatory proteins are contained in the *β*‐globulin fractions, including transferrin, complement, and ferritin [44]. Higher concentrations of transferrin are usually associated with iron deficiency anemia, resulting in a negative correlation between hemoglobin and transferrin [45]. Increasing concentrations of *β*‐globulins after the infection may be related to elevated transferrin synthesis associated with anemia in lambs infected with *H. contortus* [8]. Higher concentrations of *β*_1_‐globulins may also be caused by increased synthesis of complement components due to the activation of inflammatory processes by the infection [46]. This was clearly observable in the lamb with clinical haemonchosis, in which more than twofold higher relative concentrations of *β*_1_‐globulins, were recorded. On the other hand, the reason for the alterations in the *β*_2_‐globulin fraction in sheep is less well understood and should be further evaluated.

Sheep are a unique species among ruminants, in which *γ*‐globulins are not presented as one overall fraction on the electrophoretogram but are separated into two subfractions (*γ*_1_ and *γ*_2_). The *γ*_1_‐globulin fraction is higher compared to *γ*_2_‐globulins. Diogenes et al. [22] observed in goats infected with *H*. *contortus* an increase in the concentrations of *γ*_2_‐globulins. Similarly, Tarazona et al. [47] described in sheep with haemonchosis increased serum globulin concentrations, mainly due to increased synthesis of gamma‐globulins as a response to stimulation against parasitic infection. Furthermore, the increased concentrations of *γ*‐globulins may be attributed to the increased production of parasite‐specific immunoglobulins, especially IgA and IgE in the infected animals [48, 49]. In our study, the concentrations of *γ*_1_‐, as well as *γ*_2_‐globulins, decreased after the infection approximately till Day 42 postinfection, and the values remained lower throughout the entire observation period. Alam et al. [15] also recorded reduced serum globulin concentrations in sheep and goats with haemonchosis, which may be related to the release of antibodies from the circulation to overcome the infection directly in the abomasum. In the lamb with clinical signs of haemonchosis from our study, the relative concentrations of *γ*_1_‐and *γ*_2_‐globulins increased postinfection, and after a mild decrease of values, a further marked increase was observed, probably caused by increased synthesis of specific IgG antibodies [50]. This was seen as a diffuse increase with broad and wide peaks in the γ‐region on the electrophoretogram. These alterations in the concentrations of albumin and globulin fractions throughout the infection resulted in lambs also in changes in the A/G ratio. This reflects changes in albumin concentration in the postinfection period, especially in the period between Days 28–42, when we observed a decrease in the albumin‐to‐globulin values compared to the later postinfection period. Marked changes in the ratio of albumins and globulins in lambs with clinical signs were also reflected in significantly different and low values of the A/G ratio in the second half of the observed period.

## 5. Conclusions

In conclusion, the results of the present study showed marked alterations in the serum protein profile and distribution of serum protein fractions in lambs after the infection with *H. contortus* throughout the 107‐day postinfection period. The obtained data suggest that changes in the distribution of serum protein fractions also occur in animals without clinical signs of haemonchosis. The most intense alterations were observed in the period between Days 14 and 42 postinfection, when the concentrations of TPs and albumin decreased significantly, while the relative concentrations of *α*‐globulins increased. Our long‐lasting study showed that the values began to return to pre‐infection levels from Day 56 of follow‐up. This may reflect possible adaptation of the organism to the presence of parasites. However, the concentrations observed at the end of the evaluated postinfection period were for several parameters still outside the pre‐infection levels. On the other hand, the altered values recorded in the lamb with clinical signs of haemonchosis did not tend to return to baseline values, and there were even more significant changes and disruptions in the monitored protein profile. The present study extends the knowledge about the alterations in the serum protein profile in sheep infected with the *H*. *contortus* parasite, particularly in animals without clinically observable signs of anemia. Thus, electrophoresis of serum proteins may be useful as an additional analytical method to standard basic biochemical techniques and should be interpreted in context with the clinical state of the affected animals. On the other hand, further studies with a larger animal group in combination with hematological and special protein analyses are needed to establish which special proteins may be responsible for the alterations observed in the separated globulin fractions.

## Funding

This work was financially supported by the Vedecká Grantová Agentúra MŠVVaŠ SR a SAV (grant no. VEGA 1/0569/24).

## Conflicts of Interest

The authors declare no conflicts of interest.

## Data Availability

The data that support the findings of this study are available from the corresponding author upon reasonable request.
